# Dataset on the green consumption behaviour amongst Malaysian consumers

**DOI:** 10.1016/j.dib.2020.106302

**Published:** 2020-09-11

**Authors:** Noor Aswani Mohd Ghani, Farrah Dina Yusop, Yusniza Kamarulzaman

**Affiliations:** 1 Institute for Advanced Studies, Universiti Malaya (UM), Malaysia; 2 Faculty of Education, Universiti Malaya (UM), Malaysia; 3 Faculty of Business and Accountancy, Universiti Malaya (UM), Malaysia

**Keywords:** Sustainable consumption, Green consumerism, Pro-environmental behaviour, Green marketing

## Abstract

This dataset contains information of 375 respondents on green consumption behaviour. The questionnaire was developed using Theory of Planned Behaviour as the foundation. The variables available in the dataset are Environmental Concern (EC), Social Influence (SI), Perceived behavioural control (PBC), Consumer novelty seeking (CNS) and Green consumption behaviour (GC). In addition to the variables related to green consumption, the dataset also includes demographic and media preference information of the respondents. The data was collected via self-administered questionnaire in seven major cities in Klang Valley, namely Shah Alam, Bangsar, Petaling Jaya, Subang Jaya, Puchong, Serdang and Putrajaya. The dataset can have an important role for research in consumer behaviour towards developing green consumers.

## Specifications Table

**Table:** 

Subject	Marketing
Specific subject area	Green behaviour
Type of data	Table
How data were acquired	The data was acquired using a 36 items self-administered survey of Green consumption behaviour including additional 14 items of demographic and media preference questions.
Data format	Raw, analyzed
Parameters for data collection	The questionnaire includes 1. Demographic information such as age, gender, education level, occupation, ethnicity, marital status, personal income, number of household and work category. (9 items) 2. Media consumption preference (5 items) 3. Environmental concern (6 items) 4. Social influence (4 items) 5. Perceived behavioural control (5 items) 6. Consumer Novelty seeking (8 items) 7. Green consumption behaviour (13 items)
Description of data collection	The data was collected using a self-administered questionnaire distributed via face-to-face in high population areas in Kuala Lumpur, Petaling and Ulu Langat urban centres. The data collection took around 3 weeks to complete. A total of 430 questionnaires were distributed. However, after outliers were removed, only 375 were deemed usable for analysis.
Data source location	City: Shah Alam, Bangsar, Petaling Jaya, Subang Jaya, Puchong, Serdang and Putrajaya. Country: Malaysia
Data accessibility	Repository name: Mendeley Data Data identification number: Direct URL to data: https://data.mendeley.com/submissions/ees/edit/r5tfv3pp8k?submission_id=DIB_46952&token=d217d726-718b-417a-9d6c-8941fe29687f

## Value of the Data

• The dataset provides insights into the driving factors that influence green consumption behaviour among public.
• Data in this article will enable policy makers to make informed decision in relation to developing an action plan or policies that can entice consumers in general to partake in eco-friendly consumption.
• The dataset can be used by other researchers to compare with other data acquired from similar studies from other geographically different locations or regions.

## 1 Data Description

The dataset contains questions from five constructs of variables: Environmental concern (EC), Social influence (SI), Perceived behavioural control (PBC), Consumer novelty seeking (CNS), Green consumption behavior (GC). Definitions of each variable and references to the instrument are provided in Table 1.

**Table 1:** Variables’ dimension, definition, and adapted references of the survey instruments

Variable	Code	Definition	Adapted references of the survey instrument
Social influence	SI	The perceived social pressure to perform or not to perform a type of behaviour.	[5], [6], [7]
Perceived behavioural control	PBC	Individual's perception on how easy or difficult to perform the behaviour of interest.	[5], [6], [7]
Environmental concern	EC	Consumer's perceptions of environmental problems when making decisions.	[8,9]
Consumer novelty seeking	CNS	Refers to the openness of consumers to new products	[10]
Green consumption behaviour	GC	Consumption choice of a product that reflects an environment-related concern or motivation.	[9,11]

A total of 50 items were listed in the questionnaire with 36 items related to green consumption behaviour and 14 items related to demographics and media preference. The questionnaire was distributed to individuals aged 18 years and above, because these groups are able to make consumption decision independent from their parents [1]. A total of 430 participants responded but only 375 were relevant for further analyses after outliers were removed.

The questionnaire and SPSS codebook are provided as a supplementary file. The questionnaire was adapted from various studies incorporating concept of Consumer Innovativeness with Theory of Planned Behaviour as the basis of the research [2]. A five-point Likert scale (1 = Strongly Disagree, 2 = Disagree, 3 = Neither agree or disagree, 4 = Agree, 5 = Strongly Agree) was employed as it was found to improve respondents’ response quality and reduce fatigue while also highlights the different level in a variable [3,4]. The SI and PBC consist of four items respectively adapted from several studies [5], [6], [7]. The EC variable adapted six items from [8] and [9]. From the Consumer Innovativeness concept [10], Consumer Novelty seeking adapts eight items. Lastly the Green Consumption behaviour uses thirteen items from [9] and [11]. Additional information on media consumption preference were also collected. Skewness and Kurtosis values were computed to assess normality. Convergent validity was carried out using factor loading, Average Variance Extracted (AVE), and Composite Reliability (CR) and discriminant validity of the instruments are established by comparing the square root of all Average Variances Extracted.

## 2 Experimental design, materials, and methods

The data was collected through two sampling methods. The first method was using cluster sampling where the sampling was divided into major residential areas with the highest population density to gather random sample data [12]. Two areas were identified: (1) high populated areas which were Kuala Lumpur, Petaling and Ulu Langat; and (2) main urban areas namely Bangsar, Shah Alam, Petaling Jaya, Subang Jaya, Serdang, Puchong, and Putrajaya.

Once the location was determined, a non-probability sampling method was initiated through mall intercept method where further snowball sampling was applied. The data was analysed using SPSS software. Initial data analysis was conducted using descriptive analysis to summarize overall respondents’ demographic profiles. Then a reliability test was conducted to measure the instrument's reliability followed by factor analysis as presented in Table 2. Pearson's correlation analysis was then applied to examine the bivariate relationship amongst the variables. Finally, a regression analysis was executed to identify the relationship between the independent and dependent materials and to recognize the strongest factor in influencing the green consumption behaviour adaption.

**Table 2:** Loadings of items

Variable	Items	Before removal of item EC5 and EC6	After removal of item EC5 and EC6
Environmental concern	EC3	0.694	0.825
	EC1	0.680	0.775
	EC4	0.698	0.763
	EC2	0.732	0.682
	EC5	0.735	removed
	EC6	0.646	removed
Social Influence	SI2	−0.850	−0.845
	SI4	−0.781	−0.771
	SI1	−0.774	−0.771
	SI3	−0.771	−0.768
Perceived Behavioural Control	PBC3	−0.885	0.882
	PBC4	−0.841	0.852
	PBC2	−0.702	0.714
	PBC1	−0.680	0.670
	PBC5	−0.616	0.636
Consumer Novelty Seeking	CNS4	0.862	0.879
	CNS6	0.832	0.846
	CNS5	0.810	0.823
	CNS1	0.775	0.791
	CNS7	0.749	0.791
	CNS8	0.730	0.762
	CNS2	0.752	0.762
	CNS3	0.650	0.642
Green Consumption Behaviour	GC13	0.741	0.741
	GC12	0.739	0.739
	GC10	0.727	0.727
	GC8	0.719	0.719
	GC7	0.709	0.709
	GC11	0.704	0.704
	GC9	0.679	0.679
	GC3	0.674	0.674
	GC6	0.670	0.670
	GC4	0.662	0.662
	GC2	0.558	0.558
	GC1	0.533	0.533
	GC5	0.532	0.532

### 2.1 Reliability, normality, convergent validity and discriminant validity

The reliability of the variables was between 0.795 and 0.922, which were deemed acceptable [13]. The data normality was calculated (Table 3). Convergent validity was considered established as the values of Average Variance Extracted (AVE) is greater than 0.5 and lesser than Composite Reliability (CR). Note that although Green consumption (GC) AVE is less than 0.5, the convergent validity of the construct was acceptable as long as the CR was higher than 0.60 [14] as summarized in Table 4. Further, values of square root AVE were higher than the correlation value between items, supporting the discriminant validity of the items as showed in Table 5.

**Table 3:** Values of Skewness and Kurtosis of all items

Item	Skewness	Kurtosis	Item	Skewness	Kurtosis	Item	Skewness	Kurtosis
EC1	−.466	.041	PBC3	−.462	.357	GC2	−.707	.596
EC2	−.734	.386	PBC4	−.442	.540	GC3	−.517	.212
EC3	−.674	.653	PBC5	−.606	.999	GC4	−.552	.091
EC4	−.628	.684	CNS1	−.318	−.429	GC5	−.664	.212
EC5	−.581	−.158	CNS2	−.469	−.179	GC6	−.097	−.405
EC6	−.686	.381	CNS3	−.733	.693	GC7	−.131	−.411
SI1	−.445	.129	CNS4	−.473	.002	GC8	−.512	−.057
SI2	−.377	.233	CNS5	−.365	−.127	GC9	−.495	.070
SI3	−.318	−.201	CNS6	−.369	−.209	GC10	−.418	−.265
SI4	−.350	.364	CNS7	−.158	−.456	GC11	−.720	.388
PBC1	−.642	.241	CNS8	−.400	−.322	GC12	−.754	.306
PBC2	−.237	.046	GC1	−.739	.596	GC13	−.495	−.153

**Table 4:** Composite Reliability and Average Variance Extracted

Variable	CR	AVE	Convergent Validity	
			Did AVE > 0.5?	Did CR > AVE?
SI	0.623	0.868	Yes	Yes
CNS	0.624	0.929	Yes	Yes
EC	0.582	0.847	Yes	Yes
PBC	0.573	0.869	Yes	Yes
GC	0.448	0.912	No	Yes

**Table 5:** Values of Square root of AVE (values at the diagonal) and inter-construct correlation

Correlations					
Variable	TotalEC	TotalSI	TotalPBC	TotalCNS	TotalGC
TotalEC	**0.763**				
TotalSI	.351⁎⁎	**0.789**			
TotalPBC	.331⁎⁎	.424⁎⁎	**0.757**		
TotalCNS	.319⁎⁎	.537⁎⁎	.368⁎⁎	**0.790**	
TotalGC	.365⁎⁎	.402⁎⁎	.389⁎⁎	.604⁎⁎	**0.669**

⁎⁎ Correlation is significant at the 0.01 level (2-tailed).

## Ethics statement

This study confirms that consent was obtained from individuals who participated in the survey.

## Declaration of Competing Interest

The authors declare that they have no known competing financial interests or personal relationships which have, or could be perceived to have, influenced the work reported in this article.

## Appendix Supplementary materials

Image, application 1

Image, application 2
